# Cultural Translation and Adaptation of the Performance Analysis of Driving Ability (P‐Drive) in German for Switzerland

**DOI:** 10.1155/oti/1028430

**Published:** 2026-10-20

**Authors:** Michelle Ziswiler, Ann-Helen Patomella, Isabel Margot-Cattin

**Affiliations:** ^1^ European Master′s program ZHAW Zurich University of Applied Sciences Winterthur Switzerland zhaw.ch; ^2^ Department of Neurobiology Care Science and Society Division of Occupational Therapy Karolinska Institutet Stockholm Sweden ki.se; ^3^ Academic Primary Care Centre Stockholm Sweden; ^4^ School of Health Sciences (HESAV) Occupational Therapy Department University of Applied Sciences and Arts of Western Switzerland (HES-SO) Delémont Switzerland

**Keywords:** cultural adaptation, driving assessment, occupational therapy, on-road test, P-Drive, translation

## Abstract

**Background:**

Driving cessation can have adverse effects on an individual′s autonomy, health and well‐being. Consequently, it is essential to accurately assess the driving fitness of people with cognitive impairments. The performance analysis of driving ability (P‐Drive) has been identified as adapted for the Swiss context but is currently only available in English, Swedish, French and Norwegian. This study is aimed at translating P‐Drive into German and culturally adapt it for use in Switzerland.

**Method:**

The study comprised a two‐phase approach, namely, translation and preliminary feasibility assessment, adapted from Beaton et al. (2000), including a focus group (*n* = 6) who either work or have experience working with people with cognitive impairments and are experts on fitness‐to‐drive.

**Results:**

Adaptation of the tool included words and terminology, the inclusion of Swiss roads legal concepts, manual clarifications, scoring clarification and speed‐limit interpretation. The 25 items for assessing driving ability were retained, with a substantial modification to the scoring threshold of Item 15 that may affect its equivalence with the original item.

**Conclusion:**

A preliminary German Swiss version of P‐Drive was translated and tentatively culturally adapted to the conditions in Switzerland.

## 1. Introduction

Driving provides choice, control over daily routines and autonomy while giving a sense of independence, capability and health. It promotes social participation through community mobility [1]. Research has shown that people will outlive their driving licence, having 7–10 years of life expectancy without driving [2]. Driving cessation can negatively impact individuals′ health as it is associated with loneliness, depression, decreased self‐esteem, loss of autonomy and increased burden on the family [3–5]. It affects social engagement and increases the risks of institutionalisation.

However, driving is complex and requires automated motor skills, flexibility and quick judgement to cope with difficult traffic situations [6]. Fitness‐to‐drive, for which there are minimal medical standards, is the ability to drive a car safely in everyday traffic [7]. This depends on cognitive functions, self‐regulatory mechanisms, on‐road driving behaviours and driving characteristics, such as exposure and traffic. Underlying cognitive functions (vision and attention, executive and motor functions, awareness or metacognition and memory) can be tested using standard neuropsychological assessment procedures [8]. However, their ecological validity (mirrors real driving challenges) for assessing driving skills has been questioned, given they do not evaluate road safety risks appropriately [9, 10]. Studies examining associations between neuropsychological tests and on‐road performance report that the strength of association is too weak to correctly classify individuals as fit or unfit using off‐road tests only [11]. This has led authors to recommend using more ecological approaches, such as simulator driving or on‐road evaluations, when assessing fitness to drive [12]. On‐road assessments that test driving ability directly in traffic are therefore strongly recommended [7, 13] and are even considered the «gold standard» [14, 15].

In some countries, on‐road assessments to evaluate fitness‐to‐drive are carried out by occupational therapists in Australia, New Zealand and the United States. In European countries, including Switzerland, this is less common [16]. Occupational therapists are likely the most suitable professionals to examine and address potential connections between identified safety concerns on the road and medical conditions, including an understanding of how medical conditions and impairments can affect the ability to drive, and consequently propose viable solutions [16, 17]. A study by Korner‐Bitensky et al. [18] found that occupational therapists perceive driving‐related professional training as reasonable and are also willing to undergo further education.

On‐road driving performance assessment usually includes a closed course (e.g., in a parking lot) that allows the operational level to be safely assessed before entering traffic. Depending on the construction of the instrument, the open road evaluation then allows the investigation of the three levels of skills and control from Michon′s model [19, 20]. However, many variables influence driving, including weather conditions, users′ behaviour, road conditions and traffic, needing the professional to have expertise in observational skills. The inability to control some variables makes it difficult to standardise this type of evaluation [21]. Despite this, achieving the highest possible degree of standardisation remains essential. On‐road assessments need to be valid and reliable to support fitness‐to‐drive evaluation without compromising road safety [22]. Two recent systematic reviews examining on‐road driving tests recommended the performance analysis of driving ability (P‐Drive) as an on‐road assessment tool [14, 15].

P‐Drive assesses the quality of observable actions and the types of errors during an on‐road test on a standardised route [23]. It has been validated as a unidimensional functioning rating scale (internal validity) and contains items related to observable on‐road and behind‐the‐wheel behaviours (face validity). P‐Drive offers four levels of performance for each item and has been validated on driving simulators [24] and during on‐road tests [23]. Interrater reliability has been tested in French‐speaking Switzerland [16]. The predictive validity of P‐Drive′s raw score compared with a summed pass‐fail decision was highly reliable (0.98), revealing that P‐Drive managed to separate drivers into four levels of driving ability [22] and distinguish between those who pass and those who fail a driving evaluation. Cut‐off scores yielded high sensitivity (drivers expected to fail, failed) and high specificity (drivers expected to pass, passed) [22]. If trained occupational therapists use the instrument, P‐Drive seems to answer the need for a valid and reliable assessment tool to evaluate on‐road performance.

In German‐speaking Switzerland, no on‐road assessment tool is available in German for occupational therapists, although there is a need for and demand for them to perform fitness‐to‐drive assessments. P‐Drive was initially developed in Swedish and has been translated into several languages (Australian English, Norwegian, French and American English). To implement assessment tools in a new culture or language, simple translations are not enough [25]. Tools must also be culturally adapted for use in a different linguistic or cultural population [26]. Therefore, this study is aimed at adequately translating and cross‐culturally adapting P‐Drive into German, which fits Swiss roads′ environmental and legal driving conditions.

## 2. Materials and Methods

This study uses Beaton et al. [27] guidelines of translation and cultural adaptation, with COnsensus‐based Standards for the selection of health Measurement INstruments (COSMIN) as a complementary framework. COSMIN emphasises the importance of establishing knowledge on content validity, relevance, comprehensiveness and comprehensibility in outcome measures [28]. Beaton′s guidelines were originally developed for self‐report measures, whereas P‐Drive is an observer‐rated, performance‐based assessment tool. We retained Stages I–IV for the translation phase called Phase 1 [27].

The pretest stage was transformed into a preliminary feasibility and applicability appraisal to support cultural adaptation, because of the limited use of on‐road assessment in German‐speaking Switzerland, called Phase 2. We conducted a focus group interview with six experts, which included aspects of content validity. Before this, a face validity check [29] was done (see Figure 1). This study was approved through a clarification of responsibility process, as stated by the Ethics Committee Northwest and Central Switzerland (Req‐2022‐01518).

**Figure 1:**
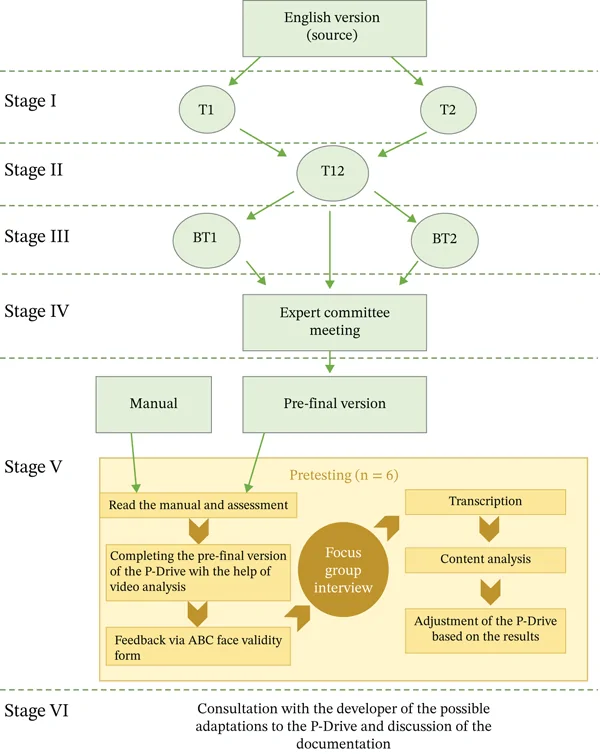
P‐Drive translation and cultural adaptation steps, including the translation phase (Phase 1: Stages I–IV) and the preliminary feasibility and applicability appraisal phase (Phase 2: Stages V–VI).

### 2.1. P‐Drive

The P‐Drive is an on‐road standardised observational assessment tool to evaluate the driving abilities of individuals with physical or cognitive impairments. It consists of 25 items organised into four categories (manoeuvre, orientate, follow regulations and attending and acting [heeding]), based on the Michon model [20] and the model of human occupation (MOHO) [30]. The rating scale has four levels of performance (4, *competent*; 3, *hesitant*; 2, *ineffective*; 1, *incompetent*), which qualify the observed ability on the road. The driving route is standardised by the sequencing of the road situation encountered (e.g., rural roads, village crossings, left turns, yields or stops, roundabouts and 30 km/h sections) by level of difficulty [14, 31, 32]. A dual‐control driving school vehicle is required, with an instructor in the front passenger seat for safety and navigation. The occupational therapist sits in the back, observing silently, taking notes and later scoring the P‐Drive.

### 2.2. Phase 1: Translating P‐Drive

The author of P‐Drive (A.H.P.) approved its translation into German and served on the expert panel. First, we conducted two translations (T1 and T2) into a target version (T‐12). The first author, an occupational therapist, knew P‐Drive and the construct it is based on (T1). The second translator was professional, naive to the construct (T2). Both translators were native German speakers and had a good command of English. Second, we merged the two translations into the T‐12 version, where discrepancies between the two translations were discussed and recorded in the written report. Third, we did two back‐translations, using the T‐12 version as a starting point. The back‐translations were done by one translator, being an occupational therapist, and the second was a professional translator. Both were aware of the construct but blinded to the original tool. They were native speakers of English and had a good knowledge of German, yielding BT1 and BT2. A written translation report was produced and archived for further use in the next stage. Multiple forward and backward translations help identify discrepancies and ensure semantic equivalence [27, 33]. Backward translations validate accuracy by detecting conceptual errors or major inconsistencies.

In the next stage, we used the five translations (T1, T2, T‐12, BT1 and BT2) and the translation reporting in an expert multidisciplinary committee to ensure adequate content for local contexts [34]. The expert committee included all four translators and the developer of P‐Drive. The expert committee worked by consensus, discussing to reach an agreement: to compare, culturally adapt and decide on the pre‐final version in German, including the adaptation of the items to Swiss road and legal driving conditions (see Figure 1).

### 2.3. Phase 2: Preliminary Feasibility and Applicability Appraisal to Support Cultural Adaptation

This stage was conducted with a group of experts (*n* = 6), using a mixed‐methods approach, combining the face validity questionnaire [29] with a focus group to provide preliminary feasibility and applicability information before considering implementation. As on‐road assessments are uncommon in the German‐speaking region, we recruited six OTs with knowledge about the fitness‐to‐drive of people with neurological disorders, mainly working in neurorehabilitation, using purposeful sampling within a convenience‐based recruitment pool. They were aged between 30 and 55 years old, had a wide variety of experiences, up to 25 years in neurorehabilitation for some. Three OTs came from the canton of Bern, the others from Zurich, Luzern and Basel. One participant has also completed training as a driving instructor and one as a neuropsychologist. All participants provided signed informed consent.

First, participants received the pre‐final version of the assessment and the accompanying manual, explaining the items and the assessment criteria with examples. They also watched a 30‐min video of a car ride, which has been used as pedagogical material for learning to score P‐Drive. None of the participants had previously viewed the pedagogical video before taking part in the pilot study. They performed an activity analysis of the video and tried out the German preversion P‐Drive, noting any ambiguities, questions and remarks. They did this individually without sharing their feedback.

Second, participants were sent Patel and Desai′s ABC face validity questionnaire (2020) to assess the face validity of the assessment, as face validity is a first step in validating an assessment tool [35, 36]. The ABC face validity questionnaire contains 10 items (scored yes or no) rating the assessment in terms of clarity, unambiguity, reasonableness and relevancy [29], listed in Table 1.

**Table 1:** Face validity form filled out by the six participants showing the rating of items (Q1–10), the criteria description and the adherence number (1–6) and ratio expressed in percentage.

Question no.	Criteria to rate	Adherence: Nb, ratio (%)
Q1	Appropriateness of grammar.	6, 100%
Q2	The clarity and unambiguity of items.	4, 67%
Q3	The correct spelling of words.	5, 83%
Q4	The correct structuring of the sentences.	5, 83%
Q5	Appropriateness of font size and space.	5, 83%
Q6	Legible printout.	5, 83%
Q7	Adequacy of instruction on the instrument.	5, 83%
Q8	The structure of the instrument in terms of construction and well thought out format.	6, 100%
Q9	Appropriateness of difficulty level of the instrument for the participants.	6, 100%
Q10	Reasonableness of items in relation to the supposed purpose of the instrument.	6, 100%

Third, participants signed an informed consent form to engage in an online focus group, which can be used when adapting an instrument that considers each country′s regional and cultural differences [37]. Its purpose was to share insights gained from reading the manual and trying out P‐Drive. The focus group addressed the manual and the assessment and discussed relevance, misunderstandings, additions, opinions and problems, focusing on the environmental and driving conditions of Swiss roads. The focus (p7 group was conducted online and lasted 3 h and 45 min. It was recorded after participants agreed to it, then transcribed by the first author. Although the focus group lasted 3 h and 45 min, short breaks were included to reduce participant fatigue. Nevertheless, the length of the session may have affected participants′ attention and engagement, particularly towards the end of the discussion, and should be considered a limitation.

### 2.4. Data Analysis

The face validity form was analysed using Patel and Desai′s [29] formula to determine the percentage of agreement for each question and the overall consent ratio. Participants added comments on where they noticed issues, found unclear or wanted to change. The first author reported this information as a preparation for the focus group.

The transcript of the focus group was analysed with a content analysis following Goold et al. [38], incorporating immersion, reduction and interpretation. The first author read the transcript and the video multiple times for familiarity (immersion). The first author inductively coded the transcript, creating a codebook using MAXQDA 2022 [39]. To strengthen the trustworthiness of the analysis, the developing codes, categories and interpretations were reviewed and discussed with the co‐authors at several stages of the analytical process. Differences in interpretation were discussed until agreement was reached, allowing the analytical framework to be refined iteratively. Some passages received multiple codes because of overlapping topics. Each code was clearly defined to ensure precision [38]. At the reduction phase, the first author, with supervision of the co‐authors, summarised memos to organise content and refined raw data, with definitions for clarity and structure. In the interpretation phase, we synthesised the memos into themes, discussing to find a consensus. Interpretive differences were treated as part of the analytic process [40]. We discussed in team meetings and examined alternative readings in relation to the data, the coding framework and the research questions. We reached consensus through reflexive dialogue and, where necessary, by revisiting the original material, leading to a coherent understanding [38] and identifying three key conditions for the cultural adaptation of the P‐Drive.

After the analysis, we adapted P‐Drive to fit the Swiss context, using memos, notes and results. The adaptation and changes were discussed with the author of P‐Drive and approved by the participants.

## 3. Findings

Table 1 shows the results of the face validity questionnaire (*n* = 6) according to Patel and Desai [29], including the 10 criteria. Calculations of the overall agreement revealed 88% agreement among participants regarding P‐Drive′s clarity, unambiguity, reasonability and relevance. However, this percentage represents one participant in disagreement. Because of the small number of participants filling out the form, no quantitative interpretation can be provided.

Although Patel & Desai [29] describe a percentage below 80 as poor, 80%–90% as substantial and 90%–100% percent as full and recommend restructuring at below 80%, revision at 80%–90% and retaining at over 90% agreement, we tentatively used the percentages as indicative of the need for adaptation, especially if items went below 67%, indicating that a third of participants disagreed.

The lowest score was for question two, the clarity and unambiguity of items, with two persons disagreeing. For this question, two participants reported back that there was some uncertainty related to the scoring system and the details of some items in P‐Drive.

The focus group interview allowed for deepening the results from the face validity assessment and continued to investigate aspects of content validity, especially in better understanding what misunderstandings (unclear and ambiguous) were apparent in the items of the translated version of P‐Drive. Three main themes were identified that led to discussions and proposals for adjustments to the German version of P‐Drive. Table 2 identifies the main adaptations and arguments for changes to the P‐Drive.

**Table 2:** Summary of main adaptations to P‐Drive, including the translation and cultural issues, and the choice made for the German version.

Original term	Translation issue	Cultural issue	Term used, correction
Item	No direct German equivalent existed that fits	The term was not consistent with Swiss occupational therapy terminology	Kompetenzen (Competencies)
Parking	Different wording in Standard German and Swiss German	Swiss road users commonly use a different term than the original Standard German word is	Parkieren (Swiss German term)
Roundabout	Several German translations possible	Swiss road traffic terminology uses a specific technical term	Kreisverkehrsplatz
Regulatory signs	Direct translation did not match Swiss traffic classifications, too long names should be avoided	Swiss law distinguishes danger, right of way and regulatory signs	Retained Vorschriftssignale and explained subcategories in the manual
Information signs	Category does not exist in Switzerland	Swiss traffic regulations use a different classification	Hinweissignale (advisory signs)
Planning	Original translation did not fully capture the intended driving behaviour	Anticipatory driving is considered essential in the Swiss context	Vorausschauen und Planen (thinking ahead and planning)
Performance	Literal translation as *Leistung* was inappropriate	The term Leistung has evaluative connotations in the Swiss context and may imply achievement rather than observed performance	Ausführung (execution)
Minor help/need help/verbal prompting (in the manual under reverse)	Distinction between support levels was unclear	Led to inconsistent interpretation among Swiss users	Additional clarification and examples in the manual
Control speed limit	Different opinions regarding the definition of speeding and the acceptable level of speed deviation	Some Swiss people consider the description of speeding to be too lenient, especially in 30 km/h zones	Exceeding the speed limit by 10% or more over an extended period is now classified as a 2‐point violation

### 3.1. Translation and Language: Not Just a Technical Issue but a Cultural One

The first and biggest challenge in the adaptation process was the choice of words and the linguistic and cultural adjustments related to language differences. Languages are spoken and written but also lived by the people, grounding the words and expressions in cultural understandings. This theme provides insights into some of the issues related to German as used in Switzerland, with specific examples.

The selection of the most fitting term for ‘item’ was discussed, as in German, no direct translation exists that accurately encompasses the meaning. Ultimately, the technical term ‘competencies’, ‘Kompetenzen’ was chosen as a suitable alternative, which the developer and the participants agreed was appropriate. Occupational therapy primarily assesses the client′s ability to competently perform certain activities, which can be considered competencies. The 25 items represent the core competencies required to drive safely on the road. Furthermore, in Eastern Switzerland, various dialects of Swiss German are spoken. Swiss German has no grammar and is mostly spoken, so Swiss people write in Standard German. During the group interview, it became apparent that some terms, words and expressions are used differently by people from Switzerland than in Germany or Austria; the two neighbouring countries using German. One example of these differences is the action of parking the car:

Interviewer: ‘Und was meinst du mit Parkieren?’ [And what do you mean by parking?] Participant 6: ‘Es ist im Hochdeutschen Parken.’ [In Standard German it′s parking.]

As a rule, the words were chosen as they are used in Swiss German, so for this example, everyone agreed on using the word ‘Parkieren’.

Roads and streets are structured and planned, dependent on habits, cultural background and procedures. Although some aspects are similar, many differences exist between countries regarding road usage. This includes signs and types of roads. For example, North American roads have very few roundabouts, whereas Europe seems to use them frequently in road design. Some countries drive on the left side, others on the right, showing a diversity of usage linked to history, habits, culture and technical terms. One of the participants was a driving instructor; he provided knowledge on these technical terms. For example, this technical knowledge was essential in deciding which term to use for roundabouts:

Genau, und in der Schweiz redet man von Kreisverkehrsplatz, das ist der Fachbegriff. [Exactly, and in Switzerland, we say traffic circles, that′s the technical term.]

In Switzerland, regulatory signs are categorised into three subgroups: ‘Gefahren‐, Vortritts‐ and Vorschriftssignale’, or in English, danger, right of way and regulatory signs, respectively. However, to prevent confusion and lengthy item names, the umbrella term ‘Vorschriftssignale’ was retained, with the subgroups described in detail in the accompanying manual. There are no information signs in Switzerland, only advisory signs, and they fit under that name. The item ‘planning’ in the German version was changed to ‘Vorausschauen und Planen’ (in English, ‘thinking ahead and planning’). Thinking ahead and planning are closely related in road traffic. Before starting a journey, knowing the traffic situation and destination is essential to determine the best route and avoid unforeseen situations. During the trip, it is important to drive with foresight. All participants agreed with the adjustment and considered it a useful addition.

### 3.2. Measuring Fitness‐to‐Drive: Levels of Performance and Comprehensibility of the Scale Levels

There were issues around the idea of performance and the use of levels to qualify fitness‐to‐drive. In the manual, the performance for Level 3 (the second‐best level in the scale) is described as ‘questionable and hesitant performance’. This was translated as ‘fragwürdige und zögerliche Leistung’. For the participants, the term ‘fragwürdig’ seemed to have a negative connotation, as expressed:

[…] I find on point three the rating questionable, hesitant performance, kind of a bit pejorative.

Because no suitable term was found during the focus group, this point was discussed with the co‐authors. ‘Occupational performance’ being a professional term with a specific definition, translating it by ‘Leistung’ was not adequate, being too similar to requirements. The adopted translation was therefore changed to ‘Ausführung’ instead.

Furthermore, participants reported ambiguity and difficulties in using the four‐level scale described in the manual. Five of the six participants had not had any education on P‐Drive. They showed more uncertainty in the assessment because they were using it for the first time, stressing the need for specific training:

Then, I think if you have more practice there, then you can divide it better than if you have done it there only once.

However, the participants′ inexperience with the P‐Drive was also a resource. They closely looked at the examples and explanations from the scale levels during the assessment and were thus able to provide detailed feedback. In the item ‘reversing’, there was confusion because all the examples in the manual talked about ‘minor help’, ‘verbal prompting’ and ‘need help’, and it was unclear what differentiated minor help from need help:

Yes, I think it is difficult to distinguish between what is meant by minor help, needed assistance and what is meant by a verbal prompting for you. Does that mean you can drive now, and no one is coming? Is that already a small request, or—yes, what is meant by that? I find it is a very difficult point.

These ambiguities are easily resolved through training.

Another discussion resulted in the scaling of the item ‘control speed limit’. Controversy arose around how much speeding is acceptable to get three points for this item. Although the English version stipulated a range of 5–10 km/h over the speed limit, Swiss participants deemed this allowance excessive, showing different cultural sensibilities, street and traffic legal structure:

For me […] 10 km/h in a 30 km zone for example is already a clear speeding. I find that unacceptable as 3 points. If the person cannot adjust their speed according to signs, in a testing situation.

Opinions varied widely, including whether a percentage should be used instead, such as 5%–10% speeding, or whether no defined limit should be specified. Some participants were very interested in a clear definition with percentages or speed, whereas others would not have accepted speeding at all. In the end, there was no unanimous opinion, but no one found the 5–10 km/h speeding acceptable, especially in low‐speed zones. The scaling was adjusted accordingly after the meeting and reviewed with the developer, who reported this change in the new manual.

### 3.3. Further Development of P‐Drive: Ideas and Suggestions

Issues or ideas emerged on how P‐Drive could be further developed. One idea was to add a not assessable column to some of the items like ‘problem‐solving’ or ‘changing gear’, as they might not always be assessable:

I think the line is great, or the column. Because an unfilled line could also just mean, I forgot… And if I then say not assessable, it′s clear, I looked at it, but I couldn′t assess.

It would help as a control in the assessment if these items were checked.

One participant suggested noting the driving test′s time. Rush hour situations or poor lighting conditions (e.g. darkening) are usually noted under the weather and road conditions on the assessment form. If the time is added, there is further information about the driving conditions, and in a second driving test, it can be seen if the time may have impacted the driving quality. The participants found adding the time a valuable enhancement to the P‐Drive.

Participants suggested adding the general car operation into the items ‘using indicator’ or ‘changing gears’. Car operation includes, for example, adjusting windshield wipers, hazard lights or high beams, if necessary. It also includes switching the car on and off and using the handbrake. Many neurological patients are quickly overwhelmed with new tasks or have difficulty operating objects correctly and planning actions. Therefore, the participants considered it useful to integrate these points.

These ideas for the further development of the P‐Drive were discussed with the developer (Stage VI). She sees a danger that, if a column ‘not assessable’ is added, it could potentially be overused. She found the idea of integrating the general car operation into P‐Drive useful but not creating a new item. P‐Drive assesses performance in terms of safety and competence in driving. The preparation for driving and using windshield wipers does not fit with the previous items and the theoretical model. So general car operation was not added in the items but in the notes below (just before the weather and road conditions), and the corresponding explanation was added to the manual.

## 4. Discussion

This study is aimed at performing a culturally appropriate translation and cross‐cultural adaptation of P‐Drive into German. The results show the outcome of the translation, including the adaptation process, and the difficulties arising in the cross‐cultural adaptation of assessment tools to diverse cultural contexts.

### 4.1. Systematic Processes for Translation and Adaptation of Assessment Tools

Translation and adaptation processes of assessment tools are specific, and there is no singular method to be used; rather, there is a need to adapt to the context and type of tool [26, 34, 41, 42] and to the possibilities available in the context. Each assessment has different characteristics and theoretical backgrounds, and each culture and language is specific, making it difficult to generalise the process. The tools must be both culturally acceptable and appropriately translated to be valid enough to be used in other countries [41]. A rigorous process with multiple steps is recommended for better translations [25], and we discuss the steps performed in this article for their adequacy.

Forward and backward translations were a form of quality control, focusing on linguistic equivalence [26]. In the subsequent meeting of the expert committee, conceptual and experiential equivalence were tested in addition to linguistic equivalence, which included the cultural meaning of terms and the relevance of the items in the assessment for the target culture [27]. Expert committees can help improve tools′ face and content validity [34].

Pretesting an assessment tool for a different culture is an essential step in evaluating the quality of the new instrument [26]. The pretesting in this study was replaced by a preliminary feasibility assessment because of the small participant group, using a mix of qualitative and quantitative methods, which is not unusual for cross‐cultural adaptation processes. Mixed methods can strengthen a study by compensating for qualitative and quantitative design weaknesses [43]. The assessment′s complexity and shortage of trained occupational therapists limited large‐scale testing. The face validity form provided light feedback on potential issues in the translated tool [29]. It built the basis for the following focus group interview, deepening aspects of especially comprehensiveness and relevance. Qualitative methods such as cognitive interviews or focus groups are just as important as the expert committee [34] and provide a valuable method of data collection in the pretest phase [26]. We opted for doing a focus group because of the shortage of OTs with experience with the P‐Drive. However, a follow‐up with cognitive interviews will be an important step in implementing the P‐Drive. Also, the developer of the tool had an important role in this process because she is aware of the theoretical background of P‐Drive and could help prevent the translated version from deviating from the original.

### 4.2. Difficulties in Adapting P‐Drive

Language and culture are indissociable because language represents the cultural background, attitude and way of thinking [44]. Adaptation to cultural conditions and values is part of the translation process. Regarding P‐Drive, the assessment takes place on the road, whose structure, regulations and practices differ from country to country. Not only does the translation of language bring misunderstanding and ambiguity but also the way the words are applied in various contexts increases this challenge for translating and adapting tools. The results show that many words had to be slightly modified to better fit the context of on‐road assessment. Linguistic difficulties can occur when, for example, two languages have non‐equivalent words [34], as was the case with the word item.

Furthermore, the language used reflects the culture of the people using it. These results need to be understood considering the use of oral dialects in Switzerland, with no written equivalent, making the transition from using a tool with people in context to a written manual and score sheet another challenge for this study. As participants came from different regions of Switzerland, they did not speak the same dialect, reflecting the diversity of the end user group for whom the translation and adaptation were done [25]. In general, it is important to have a composition of different experts in the translation and adaptation process [45]. The performance of a translated instrument for a new culture or country depends strongly on the persons involved in the translation process [25]. Regarding P‐Drive, it was particularly enriching that a driving instructor was involved, as it contains many technical terms from road traffic, and he brought a lot of expertise to the table.

The results show that some ambiguities and misunderstandings were linked to the grasp of the theoretical background in constructing the tool into four levels of performance regarding fitness‐to‐drive, applied to specific items throughout the assessment tool. The manual is there to give examples for each item and help the observer be more consistent. The findings suggest the need to improve the comprehensibility of the assessment′s structure and background to enhance its use; however, the lack of training by most of the participants could be a reason for these results. Other research [22] has indicated that training sessions are important and can help the assessor feel more confident in the evaluation. Even a short training session is sufficient to reliably educate the use of P‐Drive [16] if one understands performance levels. It is important to structure the manual in a clear and comprehensible way because, in addition to the training, it forms the basis for ensuring that different raters score as equally as possible.

When adapting tools to specific contexts, one needs to be aware of characteristics like item level, item‐to‐scale correlation and score level in the translation [27]. Results indicated that speeding of 5–10 km/h should be evaluated more strictly, thus modifying Item 15′s score level to 2 instead of 3. However, these changes in the description for the allocation of points (3 → 2) will lead to a change in the item assessed. P‐Drive is rated on a scale of safety and competence [24], and in the manual, the level of speeding should be seen as examples to guide scoring. Also, in a Rasch statistic on P‐drive in patients after stroke, Item 15 was rated as a rather simple item that showed goodness of fit to the model and underlying construct [24]. If the item, as suggested, would be scored more strictly in relation to speeding in the German version, this potentially means that this is a new item that needs further testing in relation to the previous construct. This specific change requires future psychometric examination, especially at the item‐level, interrater reliability and cross‐cultural comparability with other language versions [26].

P‐Drive has been further developed and adapted several times, and the number of driving items has varied between 20 and 27 [16, 22, 23]. However, changing the tool when adapting to a new language and context raises validity and reliability challenges. As such, there is a need to keep these translations structured and integrated within a development plan for the tool so as not to lose its quality. Conceptually, the German version remains aligned with the French P‐Drive described by Vaucher et al. [16], as both versions retain the same underlying constructs, item content and scoring framework, while adapting terminology to the respective linguistic and regional contexts.

Today, the ageing of the population in Europe, mixed with climate change and the pressure to lower CO2 emissions, adds a load to having on‐road assessment tools like P‐Drive that can support the management of fitness‐to‐drive throughout Europe and worldwide.

### 4.3. Study Limitations

The main limitation of this study is the small number of participants. As such, each participant, with their values, experience and opinions, influenced the content of the translation and adapted version of P‐Drive in German. Also, most participants had no prior knowledge of P‐Drive, which might have influenced their understanding of the scale levels. As the on‐road assessment of fitness‐to‐drive was not developed in the German‐speaking part of Switzerland, participants watched a video of an assessment to try out P‐Drive instead of doing it on‐road. These limitations make any interpretation and changes in P‐Drive tentative, showing the need for further psychometric study of P‐Drive in German [25, 34]. We did not test interrater reliability, as it had been tested in Switzerland before [16], showing good reliability. Because of the small number of participants, the face validity questionnaire percentages are unstable, with each participant contributing up to 16%, making any threshold unusable. Although participants had very diverse backgrounds and expertise, the small number is also a limitation for representing the diversity of professionals doing fitness‐to‐drive assessments in Switzerland.

Implementing P‐Drive as an on‐road assessment in the German‐speaking region of Switzerland would provide opportunities for the developer and others to continue psychometric analysis, which is also limited because of the lack of trained occupational therapists. Training needs to be developed before P‐Drive can be implemented, and further studies using P‐Drive in diverse fitness‐to‐drive assessment processes should also be planned to measure cross‐cultural equivalence.

## 5. Conclusion

This study shows how the German Swiss version of P‐Drive has been developed and culturally adapted to the context. There are limitations to this study because of the small number of participants and the shortage of on‐road assessments in this part of the country. Occupational therapists could be trained to use the P‐Drive in practice, in collaboration with driving instructors, to increase the quality and efficacy of the fitness‐to‐drive assessment processes. In this way, occupational therapists can improve their contribution to people with neurological deficits and cognitive impairments who want to drive and be integrated into the on‐road fitness‐to‐drive assessments.

## Funding

No funding was received for this manuscript.

## Conflicts of Interest

The authors declare no conflicts of interest.

## Data Availability

The data that support the findings of this study are available on request from the corresponding author. The data are not publicly available due to privacy or ethical restrictions.
